# Astrocytic activation increases blood flow in the adult olfactory bulb

**DOI:** 10.1186/s13041-024-01126-1

**Published:** 2024-08-06

**Authors:** Takashi Ogino, Masakazu Agetsuma, Masato Sawada, Hiroyuki Inada, Junichi Nabekura, Kazunobu Sawamoto

**Affiliations:** 1 Department of Developmental and Regenerative Neurobiology, Institute of Brain Science, Nagoya City University Graduate School of Medical Sciences, Nagoya, 467-8601 Japan; 2 Division of Homeostatic Development, Department of Developmental Physiology, National Institute for Physiological Sciences, Okazaki, 444-8585 Japan; 3 Institute for Quantum Life Science, National Institutes for Quantum Science and Technology (QST), Anagawa 4-9-1, Chiba Inage-ku, Chiba, 263-8555 Japan; 4 Division of Neural Development and Regeneration, National Institute for Physiological Sciences, Okazaki, 444-8585 Japan

**Keywords:** Astrocyte, DREADD, Olfactory bulb, Blood flow

## Abstract

Activation of astrocytes after sensory stimulation has been reported to be involved in increased blood flow in the central nervous system. In the present study, using a chemogenetic method to induce astrocyte activation in mice without sensory stimulation, we found that astrocytic activation led to increased blood flow in the olfactory bulb, suggesting that astrocyte activation is sufficient for increasing blood flow in the olfactory bulb. The technique established here will be useful for studying the mechanisms underlying sensory input-dependent blood flow increases.

**Supplementary Information:**

The online version contains supplementary material available at 10.1186/s13041-024-01126-1.

## Main text

In the central nervous system, astrocytes have been shown to be involved in blood flow increases induced by neuronal activity [1–3]. A recent study reported that sensory input-independent astrocytic activation by a chemogenetic method caused sustained upregulation of blood flow in the cortex [4]. In the olfactory bulb (OB), astrocyte activation has also been reported to be involved in neuronal activity-dependent blood flow increases [5, 6]. However, the mechanisms underlying the blood flow increase may be different between different brain areas [3]. Furthermore, it was unknown whether astrocyte-specific activation without olfactory input induces a blood flow increase in the OB. In the present study, we showed that astrocyte activation was sufficient for increasing blood flow in the OB.

**Fig. 1:**
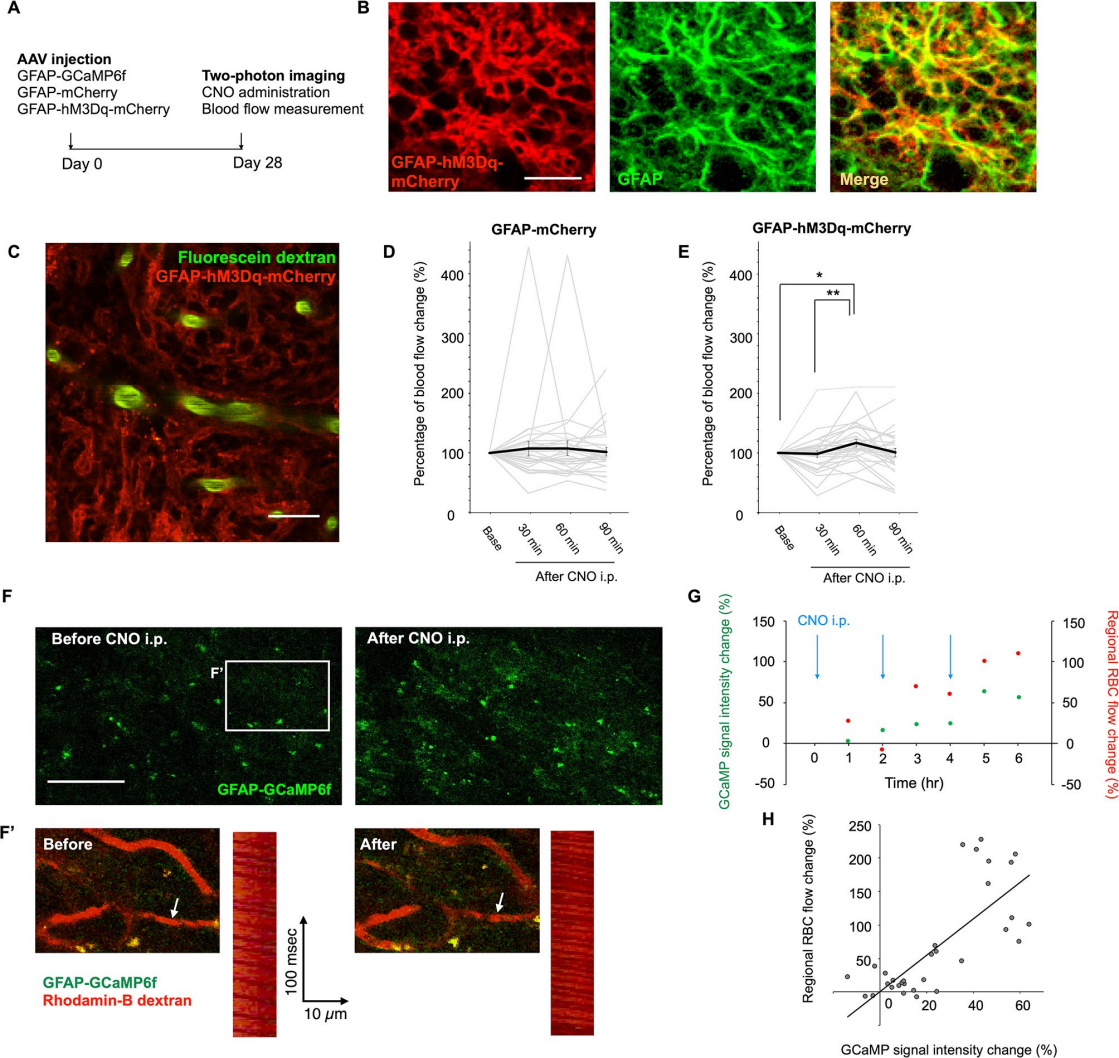
DREADD-induced astrocytic activation increases blood flow in the adult OB. **(A)** Experimental outline. **(B)** Fluorescent images in the GL. GFAP (green), mCherry (red). **(C)** A representative two-photon image in the GL. Blood vessels were visualized by intravenous injection of fluorescein dextran (green). GFAP-hM3Dq-mCherry (red). **(D**,** E)** Percentage of blood flow change after CNO administration in the GL in control (D) and DREADD (E) groups. Friedmann’s test followed by Bonferroni test was used to analyze 30 vessels from 3 mice (10, 11 and 9 vessels from each mouse) (D), and 31 vessels from 4 mice (6, 10, 6 and 9 vessels from each mouse) (E). Each data is shown as gray bars in the graphs. **(F)** Representative fluorescent images in the GL of the OB before and after CNO administration. Red blood cell (RBC) flow was recorded as two-photon line-scan images from a vessel indicated by white arrows shown in magnified images **(F’)**. GFAP-GCaMp6f (green), rhodamine-B dextran (red). Rhodamine-B dextran signals were distinguished from GFAP-hM3Dq-mCherry signals by their shape and brightness. **(G)** Representative data showing the relationship between GCaMP6f signal intensity change and regional RBC flow change. **(H)** The correlation between percentage of regional blood flow change and GCaMP6f signal intensity change in the imaging area. Pearson’s test was used to analyze 32 events obtained from multiple time points from 4 mice (8, 12, 6 and 6 events from each mouse)

CNO, clozapine N-oxide; DREADD, designer receptors exclusively activated by designer drugs: GL, glomerular layer; OB, olfactory bulb. Data are presented as the mean ± standard error of the mean (SEM). Scale bars: B, 20 μm; C, 20 μm; F, 100 μm.

In a previous study, cortical blood flow was increased by astrocyte-specific stimulation with activation of the designer receptor exclusively activated by designer drugs (DREADD), modified human M3 muscarinic receptor coupled to Gq (hM3Dq), referred to as Gq-DREADD [4]. Activation of Gq-DREADD by its selective agonist, clozapine N-oxide (CNO), causes an increase in cytosolic Ca^2+^ concentration through inositol triphosphate signaling [7–9]. To examine the role of astrocytes in blood flow changes in the OB, we introduced an adeno-associated virus (AAV) expressing hM3Dq fused with mCherry under the control of a GFAP promoter into the glomerular layer (GL) of the OB (Fig. 1A–C) of mice. We confirmed the OB-specific expression of Gq-DREADD in restricted regions around the AAV injection sites (Supplemental Fig. 1). Expression of mCherry was observed in GFAP + astrocytes in the GL (Fig. 1B). Initially, to examine the effect of astrocytic activation on blood flow, we recorded blood flow from vessel segments completely surrounded by Gq-DREADD-expressing astrocytes every 60 min after intraperitoneal injection of CNO. In the present study, we administered CNO at concentration of 1 mg/kg, at which intracellular Ca^2+^ concentration changes in astrocytes showed a continuous “plateau-like” pattern rather than an oscillation pattern as previously reported [10]. We identified blood vessel types in the GL in *NG2-DsRed* mice as previously reported [11], and the inner diameters of arterioles, venules and capillaries were 7.09–32.71 μm, 8.21–18.45 μm and 0.35–17.94 μm, respectively (Supplemental Fig. 2). The inner diameters of blood vessels from which we measured flow using two-photon imaging were smaller than 6 μm, and were thus classified as capillaries. The percentage change in blood flow was compared between the Gq-DREADD group and the control (mCherry) group. We found a transient blood flow increase in the Gq-DREADD group, but not in the control group (Fig. 1D, E), suggesting that astrocyte-specific activation increased blood flow in the GL of the OB. Next, to monitor cytosolic Ca^2+^ concentration in astrocytes in addition to blood flow changes, we injected an AAV encoding GFAP-GCaMP6f with the AAV encoding Gq-DREADD into the GL of the OB (Fig. 1A). CNO was administered every 120 min. GCaMP6f signal intensity was recorded every 30 min after the first administration of CNO, followed by blood flow recording (Fig. 1G). After CNO administration, we detected increases in both GCaMP6f signal intensity and blood flow (Fig. 1F, G). Blood flow changes were positively correlated with GCaMP6f signal intensity (Fig. 1H, *p* = 0. 000000708), suggesting that the blood flow increase after CNO administration may be caused by astrocytic activation.

Astrocyte activation was previously reported to be involved in a neuronal activity-dependent blood flow increase in the OB [5, 6]. Olfactory signals are triggered by recognition of odorants by olfactory receptors expressed in terminals of olfactory sensory neurons, and are sent to the OB neural circuits [12, 13]. These signals could also result in secondary activation of multiple types of neurons and glial cells, in addition to astrocytes in the OB [14–16]. It thus remained unknown whether astrocyte activation alone could induce blood flow changes. To investigate the role of astrocytes in blood flow increases, we chose an experimental method suitable for selective activation of astrocytes in the GL of the OB. A recent study showed that the blood flow increase could be induced by astrocyte-specific stimulation with Gq-DREADD in the cortex [4], so we used the same method in the OB. We found that astrocyte activation induced blood flow increases in the OB, which was accompanied by upregulation of cytosolic Ca^2+^ concentration in astrocytes, suggesting that astrocyte activation without stimulation of olfactory sensory neurons was sufficient for the blood flow increase in the OB. However, we could not exclude the possibility that activated astrocytes have unknown effects on olfactory sensory neurons, which have synaptic contacts with astrocytes in the GL [15]. Repetitive CNO administration every 2 h caused gradual increases in intracellular Ca^2+^ concentration in astrocytes (Fig. 1G). Considering that the serum concentration of CNO returns to its basal level within 2 h after CNO administration [17], the cumulative response may be due to CNO accumulation in the brain tissue, as previously suggested [18]. CNO administration may have widespread effects on astrocyte activation beyond the region of Gq-DREADD-expressing astrocytes through interactions among neighboring astrocytes as reported previously [19]. The blood flow increase caused by astrocytes in our study may be mediated by arteriole smooth muscle cells or capillary pericytes, which directly regulate vessel diameter [20–22]. Although we detected blood flow increases in vessels surrounded by Gq-DREADD-expressing astrocytes, it remains unknown whether activated astrocytes directly change the diameters of vessels that they surround. Activated astrocytes might have indirect effects on distant vessels. Further studies should also examine which types of vessels (arterioles or capillaries) are directly regulated by mural cells during blood flow changes caused by astrocytic activation. The technique used in this study is expected to be useful for future studies investigating the mechanism underlying blood flow changes induced by neuronal activity in the central nervous system.

## Materials and methods

### Animals

All experiments involving live animals were performed in accordance with the guidelines and regulations of Nagoya City University and the Animal Research Committee on the National Institutes of Natural Sciences. Male C57BL/6 mice (8 to 12 weeks old) were used for experiments. Wild-type mice were purchased from Japan SLC (Shizuoka, Japan). *NG2-DsRed* mice [23] were used for identification of blood vessel types. All mice were housed with free access to food and water in a 12/12-h light/dark cycle.

### Immunohistochemistry

Immunohistochemistry of mouse brain tissue was performed as previously described [24, 25]. Animals were transcardially perfused with phosphate buffered saline (PBS, pH 7.4) followed by 4% paraformaldehyde in 0.1 M phosphate buffer. The brains were removed from the skull and postfixed in the same fixative for 24 h. Coronal Sect. (50 μm thickness) were prepared using a vibratome (VT-1200 S; Leica). The sections were incubated with 10% normal donkey serum in 0.2% Triton X-100 in PBS (blocking solution) for 30 min at room temperature, followed by primary antibodies in blocking solution for 24 h at 4 °C, and finally with AlexaFluor-conjugated secondary antibodies (1:1000, Invitrogen) in the same solution for 2 h at room temperature. After staining, the sections were mounted with aqueous mounting medium (PermaFluor, Lab Vision Corporation). Z-stack images were obtained using an LSM700 confocal laser scanning microscope (Carl Zeiss) with a 20× objective lens (NA 0.8) (512 × 512 pixels, 1.25 μm per pixel, 1 μm z-step size).

The following primary antibodies were used: rabbit anti-DsRed (1:2000, 632496, Clontech), mouse anti-GFAP (1:500, G3893, Sigma) and rat anti-CD31 (1:100, 550274, BD Biosciences).

### Virus injection

The following AAVs were used: pAAV-gfaABC1D-mCherry, pAAV- gfaABC1D-GCaMP6f [26] and pAAV-gfaABC1D-hM3D(Gq)-mCherry (#50478, Addgene).

AAV injection was performed as reported previously [26]. An AAV solution (500 nL) contained in glass capillaries was stereotaxically injected into the surface of the OB in mice (4.6 mm anterior, 0.9 mm lateral to bregma, and 0.3 mm deep), 4 weeks before CNO administration using pneumatic pressure (IM 300 Microinjector, Narishige Scientific Instrument Lab, Japan).

### In vivo two-photon imaging

The open-skull surgery was performed as described previously with a modification [25]. During the operation and in vivo imaging, mice were anesthetized by inhalation of isoflurane. For the open-skull surgery, a custom-made metal plate was attached to the skull over the OB with Super-bond (Sun Medical Co., Ltd.). The skull over the dorsal OB was removed using a high-speed drill and a microhook (10065-15, Muromachi Kikai Co., Ltd.), which was followed by AAV injection into the surface of the OB as described above. Two-layer cover glasses (1 × 1 mm and 2 × 2 mm, Matsunami Glass Ind., Ltd.) were attached over the OB by Vetbond (3 M) and Super-Bond.

Two-photon imaging was performed at least 4 weeks after the surgery to minimize the effect of the skull removal and virus injection, as described previously with modification [27]. Images were acquired with a two-photon laser scanning microscope (Nikon A1R MP1, Tokyo, Japan) with a water-immersion objective lens (×25, NA 1.05, Nikon) at 950 nm. CNO (C0832, Sigma) dissolved in saline (Otsuka Pharmaceutical Factory, Inc) was administered intraperitoneally (1 mg/kg per mouse). Blood vessels were visualized by intravenous injection of Rhodamine-B dextran (D1841, Invitrogen) or fluorescein dextran (D1823, Invitrogen). Red blood cell (RBC) flow was recorded by serial line-scans as previously reported [28]. Line-shaped regions of interest were drawn along the central axis of each vessel in the square-shaped total imaging field (256 μm × 256 μm) located in the center of the imaging windows under the cover glasses. The RBC flow/sec was calculated from repetitive scans obtained over 10 s. GCaMP6f signal intensity changes were calculated by averaging the signal intensity changes detected in the total imaging field during 10 min (2 frame/s). Regional blood flow change was calculated as the average of RBC flow/sec changes in all of the vessels observed in the field.

### Statistics

Statistical analysis was performed using EZR software [29]. The normality of the data was analyzed using a Kolmogorov–Smirnov test or Shapiro–Wilk test. The equality of variance was analyzed using an *F* test. A comparison of data between three groups was performed with Friedmann’s test followed by a Bonferroni test. Correlation between two groups was confirmed by Pearson’s test. Numerical data were presented as the mean ± standard error of the mean. A *p* value < 0.05 was considered statistically significant. Significance was indicated in graphs as follows: *p < 0.05, **p < 0.01, ***p < 0.005.

### Electronic supplementary material

Below is the link to the electronic supplementary material.

**Supplemental Figure: 1 Gq-DREADD expression in the olfactory bulb**. Representative images showing the expression pattern of GFAP-hM3Dq-mCherry in the olfactory bulb. GFAP-hM3Dq-mCherry (red), CD31 (cyan). Scale bar: 500 µm

**Supplemental Figure 2: Identification of blood vessel types in the GL**. **(A)** Representative fluorescent images of the GL in NG2-DsRed mice, in which different types of vessels were identified based on the morphological differences in NG2+ mural cells. Dotted line indicates the boundary between the GL and the olfactory nerve layer (ONL). Arterioles with band-like smooth muscle cells (clear arrowheads) on the surface of the olfactory bulb were bifurcated into capillaries with pericytes in the GL. DsRed (red), CD31 (cyan). **(A’)** High magnification images from (A). Most of the vessel branches in the GL were classified as capillaries (arterioles, 0.869% ± 0.133%; capillaries, 98.6% ± 0.0778%; venules, 0.511% ± 0.146%; n = 3 mice). Pericytes had long processes (arrows) along the capillaries. Scale bar: 50 µm. GL, glomerular layer

## Data Availability

All data generated and analyzed in the study are available in the main text and supplemental files.
