# Supplementary figures and images for: Astrocytic activation increases blood flow in the adult olfactory bulb
Source: Mol Brain. 2024 Aug 6;17:52. doi: 10.1186/s13041-024-01126-1 (PMC11301997; doi:10.1186/s13041-024-01126-1)

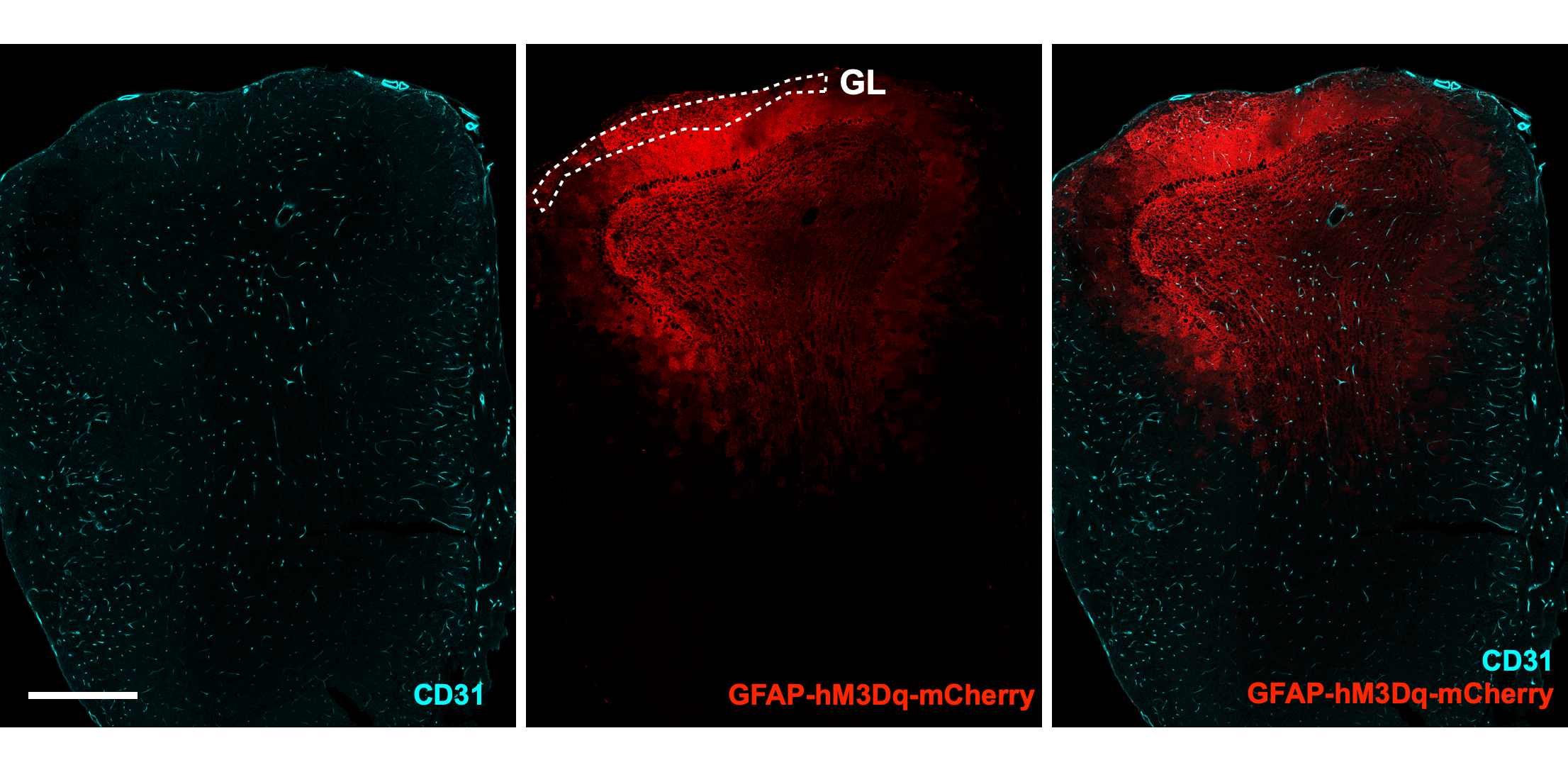

Supplement: Supplementary file 1 — Supplemental Figure: 1 Gq-DREADD expression in the olfactory bulb. Representative images showing the expression pattern of GFAP-hM3Dq-mCherry in the olfactory bulb. GFAP-hM3Dq-mCherry (red), CD31 (cyan). Scale bar: 500 µm [file 13041_2024_1126_MOESM1_ESM.tif]

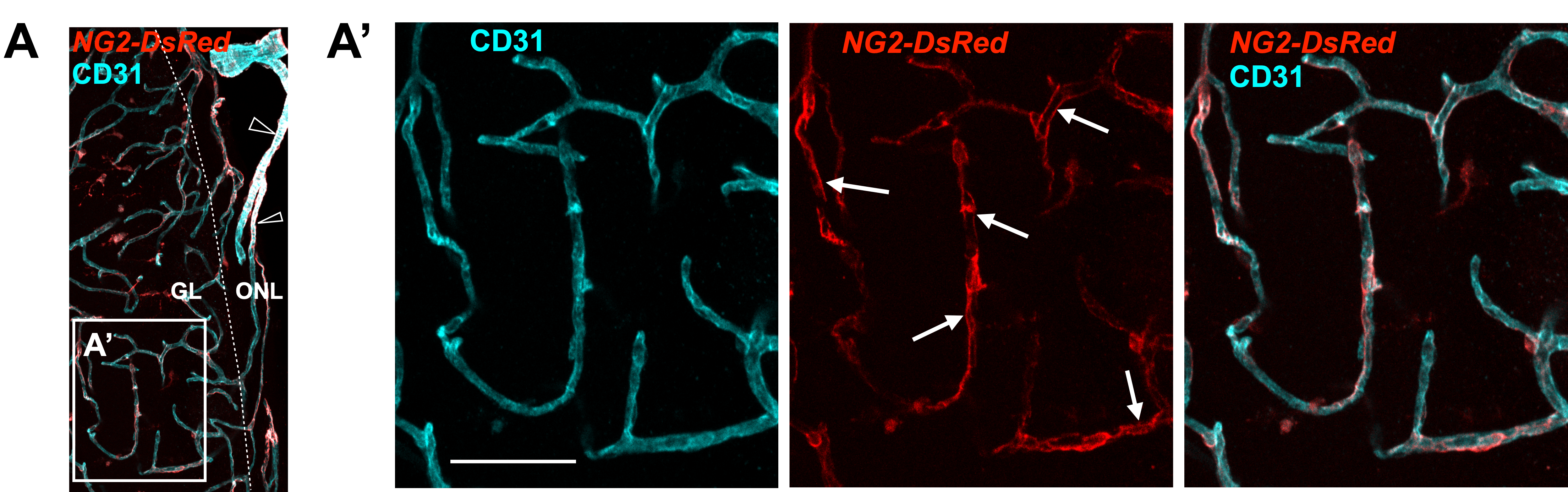

Supplement: Supplementary file 2 — Supplemental Figure 2: Identification of blood vessel types in the GL. (A) Representative fluorescent images of the GL in NG2-DsRed mice, in which different types of vessels were identified based on the morphological differences in NG2+ mural cells. Dotted line indicates the boundary between the GL and the olfactory nerve layer (ONL). Arterioles with band-like smooth muscle cells (clear arrowheads) on the surface of the olfactory bulb were bifurcated into capillaries with pericytes in the GL. DsRed (red), CD31 (cyan). (A’) High magnification images from (A). Most of the vessel branches in the GL were classified as capillaries (arterioles, 0.869% ± 0.133%; capillaries, 98.6% ± 0.0778%; venules, 0.511% ± 0.146%; n = 3 mice). Pericytes had long processes (arrows) along the capillaries. Scale bar: 50 µm. GL, glomerular layer [file 13041_2024_1126_MOESM2_ESM.tif]
